# Spousal age difference and its effect on contraceptive use among sexually active couples in Ethiopia: evidence from the 2016 Ethiopia demographic and health survey

**DOI:** 10.1186/s40834-020-00135-4

**Published:** 2020-11-23

**Authors:** Sena Belina Kitila, Yonas Biratu Terfa, Adugna Olani Akuma, Ayantu Kebede Olika, Alemi Kebede Olika

**Affiliations:** 1 Faculty of Health Sciences, School of Nursing and Midwifery, Jimma University Institute of Health Science, Addis Abeba, Ethiopia; 2 Department of Epidemiology, Faculty of Public Health, Jimma University Institute of Health, Addis Abeba, Ethiopia; 3 Population and Family Health Department, Faculty of Public Health, Jimma University Institute of Health Science, Addis Abeba, Ethiopia

**Keywords:** Spousal, Age difference, Contraceptive, Couples, Ethiopia

## Abstract

**Background:**

Age difference among spouses can be considered as an indicator of the nature of the marital bond, and influences the couple’s fertility expectations. The age difference is one of the features of the traditional African marriage system. However, the likelihood that women use of contraceptives and spousal age differences is not well studied. Thus, this study was to examine the spousal age difference on contraceptive use.

**Objective:**

This study was aimed to examine spousal age differences and its effect on contraceptive use among sexually active couples in Ethiopia.

**Methods:**

The related variables for this study were extracted from Ethiopian Demographic and Health Survey 2016 data. IBM SPSS statistics version 20 software was used for analysis. Logistic regression was conducted to see the association between spousal age difference and contraceptive use. All analyses were adjusted for sample weights.

**Results:**

Out of the 7268 selected women for contraceptive usage questions, one fourth (25.3%) of them were between ages 25 and 29 and in almost all 7061 (98.4%) of them there was spousal age differences, 1555 (21.4%) of them were from poor socioeconomic group. Nearly all 7184 (98.8%) of them knew contraceptive method. However, among those sexually active in the last 4 weeks only two in five (41.2%) were using a contraceptive method.

Spousal age difference was found to be significant factor and women older than their spouses were (AOR: 1.771, 95%CI: 1.276, 2.459) more likely and women having spouse’s age difference greater than 10 years were 1.2% (AOR: .988, 95%CI: .848, 1.150) less likely to use contraception compared to those age difference is ten or less than years respectively.

Also, women who were living in urban areas (AOR: 1.482, 95%CI: 1.161 to 1.890), current working status (AOR: 1.170; 95%CI: 1.033 to 1.325), from richest economic category (AOR: 2.560; 95%CI: 2.000 to 3.278) husband’s education, couples’ fertility preference (AOR: 1.233; 95%CI: 1.070 to 1.420) were contraception use predictors. Similarly, being Muslim by religion (AOR: .579 95%CI: 0.496 to 0.675) and husband based decision for their health care use were (AOR: .847, CI: .729 to .985) less likely to use contraception.

**Conclusion and recommendation:**

This study found association between spousal age differences and contraceptive use. Similarly, women’s age, age difference, place of residence, religion, current working status, socioeconomic, husband’s education, living children and current pregnancy, the couples’ fertility preference and who decides on health care use were found to be predictors of contraceptive use. Strengthening strategies for improving women’s educational status, socio-economic and demographic that will help to limit the age differences and improve contraceptive use. Further study, including qualitative is recommended to dig out the why components and better understand this finding.

## Introduction

The age difference among spouses can be considered as an indicator of the charactertics of the marital bond, and influences the couple’s fertility expectations. At the individual level, a large age difference makes the distance between the spouses, since age is vital for forming a relationship of subordination, and it generates a generational and cultural gap between spouses those are at different phases in their life cycle and who had different experiences and understanding of pre-marital life [1].

A big age difference between couples is one of the features of the traditional African marriage system [1] and this age difference between spouses influence marital stability, marital satisfaction, family size preference and contraceptive use [2].

Also the likelihood that women use contraceptives may be reduced when they are significantly younger than their husband, because such age differences are often accompanied by incongruences in social position, resources and life experiences, which may make marital relationships integrally unequal [3].

The study conducted in India to assess the influence of the age gap between couples on contraception and fertility indicated that age at marriage for husband and wife, the age gap between spouses, desire for additional child, household structure, residence, education, religion, caste, standard of living and mass media exposure affect the use of contraception and fertility behavior [4].

The secondary analysis of the Tanzania Demographic Health Survey (TDHS), 2010 indicated that women empowerment, male-female age difference, child desire, partners education and advice given at health care facilities on family planning were predictors of modern contraceptive use [5].

The analysis of 2008/09 Kenya Demographic and Health Survey (DHS) to look at the effect of age difference on contraception use indicated that the spousal age difference has no association with contraception use [6].

The analysis of 2014 Kenya Demographic and Health Survey (DHS) to look at the determinants of modern contraceptive use found that region of residence, marital status, religion, wealth, interaction with a health care provider, fertility preference, number of sexual partners and access to media were all significantly associated with modern contraceptive use [7].

The cross sectional study conducted in Urban Kenya to see the Couple Characteristics and Contraceptive use showed spouses’ education, religion and income were correlated with contraceptive use [8].

The analysis of 2008 Nigeria Demographic and Health Survey (DHS) to assess the age difference between partners and contraceptive use indicated that there was no association between age differences between partners’ and women’s contraceptive use but household wealth and regional differences were predictors [3].

The cross sectional study conducted in Jimma zone to assess spousal discordance on fertility preference and its effect on contraceptive practice indicated that wife’s age, husband’s desire for more children, husband’s favorable attitude towards family planning and husband’s preparedness to accept his wife’s use of contraceptives without his consent are significant contributors for contraceptive use [9]. Due to the nature of human sexual reproduction, men more attracted to women who appear to be healthy and fertile. Thus age is an important visible cue of a woman’s fertility, given that women have a limited reproductive window. This helps to explain why younger men’s age preferences are not as pronounced as those of older men. That is, preferring younger and younger partners are more likely to result in successful reproduction [10].

All women who want to prevent pregnancy are able to obtain and use modern contraceptives, that they are given their choice otherwise impair their health and limit their prospects for productive participation in society. Yet, unplanned pregnancies among women happen despite the best of contraceptive intentions [11–13], (https://www.worldometers.info/world-population/population-by-country/). Hence, this study was aimed to examine the spousal age difference and its effect on contraceptive use among married couples at county level.

## Methodology

### Study setting

Ethiopia is, the largest nation in the Horn of Africa, and country bounded by Eritrea from the north, Sudan and South Sudan from the West, Kenya from the South, and Somalia and Djibouti from the East. The capital city of the country is Addis Ababa. Ethiopia is the second most populous country in Africa and 12th in the world with an estimated population of 112.73 million. The male to female ratio is almost 1:1 (https://www.worldometers.info/world-population/population-by-country/). Women of childbearing age account for about 57% of the female population and 30% of the total population. Hence, the contraceptive prevalence rate (CPR) for currently married women age 15–49 was 36% and unmet need for family planning among currently married women is 22% [14].

### Sampling

The data for this study was obtained from the 2016 Ethiopian Demographic and Health Survey (EDHS). The sampling frame for the study was a complete list of 84,915 enumeration areas (EAs) created for the 2007 population and housing census [PHC]. EDHS typically adopt a two-stage sample design. Each region was stratified into urban and rural areas, which yielded 21 sampling strata. Samples of Enumeration areas [EAs] were selected independently in each stratum in two stages. The first stage involves randomly selecting clusters, EAs. Two hundred two, 202 urban and 443 rural clusters were selected. At the second stage a systematic sample of households is drawn from a listing of households in each of the sampled clusters-28 households per each cluster were selected.

#### Data collection

Women between 15 and 49 years who were either permanent residents of the selected households or visitors who stayed in the household the night before the survey were eligible to be interviewed and one woman per household was selected. The EDHS data collection tools were divided into five sets: The Household Questionnaire, the Woman’s Questionnaire, the Man’s Questionnaire, the Biomarker Questionnaire, and the Health Facility Questionnaire. Data on contraceptive use was collected using The Woman’s Questionnaire.

Interviewers selected for the survey used tablet computers to record responses during the interviews. The tablets were equipped with Bluetooth technology to enable remote electronic transfer of files. Transfer of assignment sheets from team supervisor to interviewers and transfer of completed questionnaires from interviewers to the supervisor were performed electronically through the CSPro software [15].

### Variables

**Dependent variables:** contraceptive use (Yes/No).

**Independent variables**: Age category, age difference (Age difference = Husband’s Age minus Wife’s Age), place of residence, religion, region, sex of household head, wealth status, women’s level of education, husband/ partner’s education level, respondent’s current working condition, knowledge of any method, recent sexual activity, fertility preference of the husband and fertility preference of the women were taken as independent variables.

### Data processing and analysis

Relevant variables for this study were extracted from Ethiopian Demographic and Health Survey [EDHS] 2016 data. We used IBM SPSS statistics version 20 software for analysis. The data were described using frequency and percentage. Bivariate logistic regression was conducted to examine the crude association between spousal age difference and contraceptive use. Finally, multiple logistic regressions were conducted to identify independent predictors of contraceptive use. Variables with *p* value less than 0.05 were considered as significantly associated with contraceptive use. All analyses were adjusted for clusters and sample weights.

## Results

### Description of study participants

This section describes the distribution of women (*N* = 7268) by their selected background characteristics using the EDHS data set. The highest proportion 1841 (25.3%) of women were within age groups of 25 and 29 years. Almost two fifth 2897 (39.9%) of the women were from the Oromia regional state. Most of the women 6237(85.8%), were residents of rural areas. Three thousand and sixty-four (42.2%) were Orthodox by religion followed by Muslim 2442 (33.6%). Almost all 6969(95.9%) of the households were headed by males. Only 454 (6.2%) and 247 (3.4%) of the husbands and wives had attained their higher education respectively. Nearly one fifth 1555 (21.4%) of them were from poor socioeconomic group (Table 1).

**Table 1:** Distribution of women by their selected background characteristics, 2016 EDHS

Variable	Category	Frequency(***n*** = 7268)	Percent
Age	15–19	419	5.8
	20–24	1272	17.5
	25–29	1841	25.3
	30–34	1468	20.2
	35–39	1169	16.1
	40–44	681	9.4
	45–49	417	5.7
Region	Oromia	2897	39.9
	Amhara	1879	25.9
	SNNPR	1526	21.0
	Tigray	408	5.6
	Addis Adaba	214	2.9
	Somali	163	2.2
	Others ^a^	181	2.5
Residence	Urban	1031	14.2
	Rural	6237	85.8
Religion	Orthodox	3064	42.2
	Muslim	2442	33.6
	Protestant	1612	22.2
	Other^b^	150	2
Sex of household head	Male	6969	95.9
	Female	299	4.1
Husband/partner’s education level	No education	3229	44.4
	Primary	2908	40.0
	Secondary	639	8.8
	Higher	454	6.2
	Don’t know	38	.5
Highest educational level women	No education	4465	61.4
	Primary	2131	29.3
	Secondary	425	5.8
	Higher	247	3.4
Respondent currently working	No	5076	69.8
	Yes	2192	30.2
Wealth Index	Poorest	1280	17.6
	Poorer	1555	21.4
	Middle	1514	20.8
	Richer	1504	20.7
	Richest	1414	19.5

Others ^a^ Afar, Benishangul, Gambela, Harari and Dire Dawa

Other^b^ Catholic, Traditional, Other

### Experience of contraceptive use

Regarding knowledge of contraceptive method almost all 7189 (98.9%) of them knew the contraceptive methods. On the other hand, only three in five 4438 (61.1%) of them had history of contraceptive use to delay or avoid getting pregnant. As to the sexual practice in the last 4 weeks almost nine in ten (87.2%) were active. However, only two in five 2608 (41.2%) were using contraceptive method currently. Also 1692(45.4%) do not intend to use the contraceptive. The majority 4619 (63.6%) and 4142 (57.0%) of the husbands and wives have fertility preference respectively. More than one fifth 1640 (22.6%) them have six and above children (Table 2).

**Table 2:** Distribution of women by their experience of contraceptive use, 2016 EDHS

Variable	Category	Frequency(***n*** = 7268)	Percent
Knowledge of any method	Knows no method	79	1.1
	Knows methods	7189	98.9
Ever used contraceptive	No (Not used outside and in calendar)	2830	38.9
	Yes (used outside and in calendar)	4438	61.1
Recent sexual activity	Active in last 4 weeks	6335	87.2
	Not active in last 4 weeks	932	12.8
Current use of contraceptive(*n* = 6335)	Not using	3728	58.8
	Using	2608	41.2
Intention to use (*n* = 3728)	Use later	1942	52.1
	Unsure about use	94	2.5
	Does not intend	1692	45.4
Fertility preference of the Husband	Have another	4619	63.6
	Undecided	222	3.1
	No more	2265	31.2
	Others ^a^	162	2.3
Fertility preference of the women	Have another	4142	57.0
	Undecided	386	5.3
	No more	2622	36.1
	Others ^a^	118	1.7
Living children and current pregnancy	No child	456	6.3
	One Child	1105	15.2
	Two Children	1135	15.6
	Three Children	1107	15.2
	Four Children	994	13.7
	Five Children	831	11.4
	Six and above Children	1640	22.6

^a^ Sterilized (respondent or partner and Declared infecund

### Decision making and Age difference

Decision on contraceptive use was made a jointly 2108 (75.6%), likewise there was joint decision on health care aspects among 5163(71.0%). However, in matters concerning household expenditure, one in five 318 (22.9%) respondents were reported independent decision making. The age difference is calculated by using husband’s age minus wife’s age and further categorized negative (Age of wife is greater), equal greater than 10 years and less than 10 years to see the implication of age deference indeed, age-gap by more than 10 years perceive substantially more social disapproval regarding their relationship than do couples with only a minimal or no age gap. More than three fourth (78.6%) of husbands’ were in age difference less than 10 year compared to their wife. (Table 3).

**Table 3:** Distribution of women by their age difference and decision making, 2016 EDHS

Variable	Category	Frequency	Percent
Decision maker for using contraception (*n* = 2788)	Mainly respondent	523	18.8
	Mainly husband, partner	154	5.5
	Joint decision	2108	75.6
	Other	3	.1
Person who usually decides on respondent’s health care	Respondent alone	760	10.5
	Respondent and husband/partner	5163	71.0
	Husband/partner alone	1322	18.2
	Other	22	.3
Who decides how to spend respondent’s earnings(*n* = 1388)	Respondent alone	318	22.9
	Respondent and husband/partner	949	68.4
	Husband/partner alone	121	8.7
Age of the husbands’ to women’s	Negative (Age of wife is greater)	208	2.9
	Age of husband is equal to that of wife	158	2.2
	Age of husband is greater by ≤10 years	5710	78.6
	Age of husband is greater by >10 years	1192	16.4

### Association between spouses age difference and contraceptive use

Women age, age difference, place of residence, religion, current working status, socioeconomic, husband’s education, women’s education, living children and current pregnancy, the couples’ fertility preference and who decides on health care use were candidate variables for multiple logistic regression. And in the final model women age, age difference, place of residence, religion, current working status, socioeconomic, husband’s education, living children and current pregnancy, the couples’ fertility preference and who decides on health care use were remained statistically significant determinants of contraceptive use.

A spousal age difference was found to be a significant factor affecting the use of contraceptives. Women, older than their spouses were 1.77 times [AOR = 1.771, CI (1.276, 2.459) more likely to use contraceptives compared to those women, their husband age is greater than their age by ten or less years. Also, women having older spouse’s age differences greater than 10 years were by 1.2% [AOR = .988, CI, .848, 1.150] less likely to use contraception compared with those women and husband age differences is ten or less than years. Also women who were living in urban area were 1.482 times (AOR = 1.482, CI, 1.161 to 1.890) more likely to use contraception compared to a woman living in rural area.

The probability of contraception use was 1.170 times (AOR: 1.170; 95%CI: 1.033 to 1.325) more likely among women who were currently working compared to women who were not working at the time of the survey. Being women from the richest household economic category were 2.560 times more likely to use contraception compared with women from the poorest households (AOR: 2.560; 95%CI: 2.000 to 3.278).

Similarly, the partner’s education level was found to be an important determinant of contraceptive use and husband with primary and higher education were 1.042 and 1.282 times more likely to use contraceptives, respectively, than women with no formal education. In addition, those whose husbands had no more fertility preference were 1.233 times (AOR: 1.233; 95%CI: 1.070 to 1.420) more likely to use contraceptives compared with those who have no fertility preference.

Moreover, contraceptive use was 15.1% less likely among women decision make for their health care use was their husband alone compared to women who made the decision by themselves (AOR: .847, CI: .729 to .985) (Table 4).

**Table 4:** Bivariate and multivariate logistic regression model showing predictors of spouses’ age difference and contraceptive use, 2016 EDHS

Variable and its category		COR [95% CI]	AOR [95% CI]
Women age classification	15–19	.713(.564, 900)	.892 (.665, 1.197)
	20–24	.890 (.764, 1.037)	.941 (.784, 1.128)
	25–29	1	
	30–34	.825 (.711, 956)	.886 (.746, 1.052)
	35–39	.773 (.659, 907)	.882 (.723, 1.077)
	40–44	.805 (.666, 974)	.877 (.687, 1.120)
	45–49	.348 (.266, 454)	.455 (.326, 636)*
Age difference	Negative (Age of wife is greater)	1.506 (1.131, 2.006)	1.771 (1.276, 2.459)*
	Equal to that of wife	.960 (.677, 1.361)	.790 (.542, 1.153)
	Age of husband greater by ≤10 years	1	
	Age of husband greater by 10 years	.963 (.839, 1.106)	.988 (.848, 1.150)
Type of place of residence	Urban	2.612 (2.258, 3.022)	1.482 (1.161, 1.890)*
	Rural	1	1
Religion	Orthodox	1	
	Protestant	.818 (.719, 930)	.966 (.808, 1.155)
	Muslim	.359 (.317, 405)	.579 (.496, 675) *
	Other	.618 (.419, .912)	.944 (.611, 1.459)
Current working status	Not working	1	
	Working	1.506 (1.352, 1.677)	1.170 (1.033, 1.325) *
Wealth Index	Poorest	1	1
	Poorer	1.967 (1.640, 2.358)	1.796 (1.479, 2.180) *
	Middle	2.662 (2.224, 3.186)	2.314 (1.906, 2.809) *
	Richer	2.854 (2.385, 3.415)	2.454 (2.015, 2.988) *
	Richest	4.668 (3.891, 5.601)	2.560 (2.000, 3.278) *
Husband/partner’s education level	No education	1	
	Primary	1.321 (1.183, 1.475)	1.158 (1.013, 1.323) *
	Secondary	1.684 (1.404, 2.021)	1.040 (.823, 1.316)
	Higher	3.120 (2.505, 3.885)	1.282 (.941, 1.746)
	Don’t know	1.043 (.524, 2.079)	.828 (.397, 1.723)
Highest educational level women	No education	1	
	Primary	1.474 (1.318, 1.649)	1.042 (.904, 1.202)
	Secondary	2.524 (2.033, 3.134)	1.142 (.859, 1.518)
	Higher	3.439 (2.579, 4.585)	1.175 (.786, 1.756)
Living children and current pregnancy	No child	2.013 (1.611, 2.516)	1.674 (1.193, 2.349) *
	One Child	2.155 (1.815, 2.560)	2.148 (1.636, 2.820) *
	Two Children	2.795 (2.358, 3.313)	2.669 (2.093, 3.404) *
	Three Children	1.978 (1.666, 2.348)	1.844 (1.478, 2.302) *
	Four Children	1.793 (1.501, 2.141)	1.681 (1.362, 2.076) *
	Five Children	1.318 (1.088, 1.598)	1.331 (1.073,1.650) *
	Six and above Children	1	
Fertility preference of the women	Have another	1	1
	Undecided	.604 (.469, 778)	.813 (.614, 1.077)
	No more	1.123 (1.010, 1.248)	1.603 (1.391, 1.849) *
	Others	.386 (.235, 634)	.118 (.039,.353) *
Fertility preference of the Husband	Have another	1	1
	Undecided	.957 (.710, 1.289)	1.229 (.870, 1.736)
	No more	1.084 (.972, 1.209)	1.233 (1.070, 1.420) *
	Sterilized	.342 (.176, 666)	.231 (.088,.603) *
	Declared infecund	.299 (.163, 547)	.430 (.220, 841) *
Who decides on health care	Respondent alone	1.021 (.863,1.208)	.900 (.745, 1.087)
	Respondent and husband/partner	1	
	Husband/partner alone	.647 (.564,.742)	847 (.729,.985) *
	Other	5.988 (2.019, 17.765)	15.235 (4.456, 52.085)*

*Significant *p* ≤ 0.05

## Discussion

This analysis shows that just nearly nine in ten (87.2%) of the women were sexually active during the last 4 weeks of the survey period. However, only two in five (41.2%) were reported the contraceptive use during the study. This implies that three fifth of them were not using the contraceptives, although, almost all (98.8%) of them knew the contraceptive methods. This indicated that besides the knowledge factor, as a number of socio-economic, demographic and cultural factors contribute to the contraceptive use [4].

This study further revealed the relationship between the spouses’ age difference and contraceptive use. And the age difference of the spouses was found to be a significant factor affecting the use of contraceptives, women whose husbands’ age was greater than their age was less likely to use a contraceptive compared with those who were older than their husbands.

Also, in the social and psychological literatures, age-gap carries some meaning for partners in any relationship and differences beyond 10 years appear to be regarded as non-normative, this leads to marital satisfaction declines. Substantially leads to more social disapproval regarding their relationship than does couples with only a minimal or no age gap [10, 16]. The finding was also in line with the study conducted in India, where the age difference between spouses influence marital stability, marital satisfaction, family size preference and contraceptive use [2]. This may be due to when the husbands are older than their wives; there is possible disparity in social position, resources and life experiences. Thus, women with older husbands, therefore tend to have lesser autonomy in the socioeconomic sphere like education, economic activity, access to resources, and in interpersonal and family relationships, such as freedom of speech, freedom to travel, decision-making power regarding childbearing and contraceptive use [1, 3, 17].

Similarly, women age, participant’s residence, religion, current working status, socioeconomic, husband’s education, living children and current pregnancy, the couples’ fertility preference and who decides on health care use were found to be important contributing factors for contraceptive use [4].

This finding is consistent with the analysis from 2010 Tanzania Demographic Health Survey (TDHS), 2014 Kenya Demographic and Health Survey (DHS), the cross sectional study conducted in Urban Kenya, Turkey where women empowerment, male-female age difference, partners education and advice given at health care facilities on family planning, region of residence, marital status, religion, wealth, interaction with a health care provider, fertility preference, number of sexual partners and access to media were found to be predictors of modern contraceptive use [6, 8, 9, 17].

Also consistent with the study conducted in India, where the age gap between couples contraception use and fertility influenced by age at marriage for husband and wife, the age gap between spouses, desire for additional child, household structure, residence, education, religion, standard of living and mass media exposure [4]. This could be due to the reason that the more the husband is educated, the more access to media, have stable household structure, health seeking behavior and he would be aware of the benefits of contraceptive, less desire for more child as development itself is the best contraceptive so that he encouraged his wife to use contraceptive.

The age difference between spouses may be one of the sign for woman’ s autonomy, rural women usually have less access than urban women to contraception, information about contraceptive methods and access to health care that leads to less use of the contraceptive, there are also differences in contraceptive use by education, as the level of education is likely to affect their knowledge of contraceptive methods, its side effects, perceptions about contraception and factors like socio-economic, demographic and cultural connotations these reasons might be among the contributing factors to contraceptive use.

However, this finding was unlike the analysis of 2008/09 Kenya Demographic and Health Survey (DHS) and 2008 Nigeria Demographic and Health Survey (DHS) where the spousal age difference has no association with contraception use but household wealth and regional differences were predictors [3, 6].

## Conclusion

This study found an association between spousal age differences and women’s contraceptive use. Similarly, women age, age difference, participant’s residence, religion, current working status, socioeconomic, husband’s education, living children and current pregnancy, the couples’ fertility preference and who decides on health care use were found to be predictors of contraceptive use. Strengthening strategies for improving women’s educational status, socio-economic and demographic that will help to limit the age differences and improve contraceptive use. Further study, including the use of qualitative study is recommended to dig out the why components and better understand this finding.

## Data Availability

The raw data available at https://dhsprogram.com/publications/publication-fr328-dhs-final-reports.cfm

## Abbreviations

EDHS: Ethiopian Demographic and Health Survey
SPSS: Statistical Package for the Social Sciences
AOR: Adjusted Odd ratio
CI: Confidence Interval
TDHS: Tanzania Demographic Health Survey
DHS: Demographic and Health Survey
EAs: Enumeration areas
PHC: Population and housing census
